# Disease Risk from Human–Environment Interactions: Environment and Development Economics for Joint Conservation-Health Policy

**DOI:** 10.1007/s10640-020-00449-6

**Published:** 2020-07-09

**Authors:** Heidi J. Albers, Katherine D. Lee, Jennifer R. Rushlow, Carlos Zambrana-Torrselio

**Affiliations:** 1 Depertment of Economics, University of Wyoming, 1000 E University Ave., Laramie, WY 82071 USA; 2 Department of Agricultural Economics and Rural Sociology, University of Idaho, Moscow, ID 83844 USA; 3 EcoHealth Alliance, New York, NY 10001 USA

**Keywords:** Bats, Deforestation, Disease, Fragmentation, Land use, Pathogen spillover, Wildlife markets, Zoonoses

## Abstract

Emergence of COVID-19 joins a collection of evidence that local and global health are influenced by human interactions with the natural environment. Frameworks that simultaneously model decisions to interact with natural systems and environmental mechanisms of zoonotic disease spread allow for identification of policy levers to mitigate disease risk and promote conservation. Here, we highlight opportunities to broaden existing conservation economics frameworks that represent human behavior to include disease transmission in order to inform conservation-disease risk policy. Using examples from wildlife markets and forest extraction, we call for environment, resource, and development economists to develop and analyze empirically-grounded models of people’s decisions about interacting with the environment, with particular attention to LMIC settings and ecological-epidemiological risk factors. Integrating the decisions that drive human–environment interactions with ecological and epidemiological research in an interdisciplinary approach to understanding pathogen transmission will inform policy needed to improve both conservation and disease spread outcomes.

## Introduction

Soon after the discovery of SARS-2-COV, COVID-19 became a pandemic event that spread worldwide, infected millions of people, and created unprecedented economic impact (WHO 2020a; Guan et al. 2020). Current evidence suggests that COVID-19 resulted from a zoonotic pathogen “spillover” event—transmission of a pathogen from wildlife to humans (Andersen et al. 2020). The majority (~ 60%) of the emerging infectious diseases (EIDs) since 1940 had an animal origin with most of these (> 70%) caused by pathogens associated with wildlife (Jones et al. 2008). Although pandemics are rare, zoonotic disease incidence and novel spillover events cause frequent and serious health, social, and economic losses in tropical low and middle income countries (LMICs) (Morse et al. 2012). As COVID-19 spread around the globe, several countries in Latin America experienced an extreme outbreak of Dengue and other vector-borne diseases (Lorenz et al. 2020) and the Democratic Republic of the Congo endured their eleventh Ebola outbreak since 1976 (WHO 2020b). Because zoonotic spillover events occur in human-animal interfaces, policy to address such disease risks requires interdisciplinary analysis of the epidemiological, environmental, and human processes occurring on the landscape.

Data analyses find that disease emergence and biodiversity loss share common anthropogenic drivers: land use change, habitat fragmentation, expansion of the agriculture frontier, and wildlife harvest and trade (Patz et al. 2004; Wolfe et al. 2005; Loh et al. 2015; Huong et al. 2020). Given that people’s decisions are the root of these drivers, the choices people make about interactions with the environment influence both conservation outcomes and infectious disease risk (Murray and Daszak 2013). In many LMICs, human–environment interactions occur during activities that are integral to daily life and are a function of specific socio-economic and institutional settings (Albers and Robinson 2013; Nielsen et al. 2017). Establishing policy to increase conservation and reduce disease risk requires understanding both people’s decisions—including land use, resource extraction, and market activities—within their context, and how environment-human interactions lead to disease risk.

Environment, resource, and development economists explore models and data to determine why and how humans interact with the environment in LMICs, which informs conservation policy. Here, we review how these economic frameworks capture—or do not capture—drivers and characteristics of the human–environment interaction, while reflecting the natural and socio-institutional settings of LMICs. We then propose how modeling frameworks can be expanded to incorporate the disease risk posed by that interaction to inform needed socio-enviro-epidemiological research and policy analysis, using an iterative process of data collection and modelling in an interdisciplinary setting. Finally, we discuss how expanded models can provide insight into behavioral drivers of risk and inform design of policy for two general types of pathogen transmission pathway from wildlife to humans: direct—contacting a pathogen through hunting, marketing, or consumption of wildlife, with an example from Indonesian wildlife markets; and indirect—exposure to pathogens while undertaking livelihood activities, with an example of malaria transmission. Filling this research gap will address calls from many conservation and health actors to incorporate human dimensions of disease risk into joint conservation-health policy (Romanelli et al. 2014; Díaz et al. 2015; Whitmee et al. 2015; WHO 2015).

## Conservation Economics in LMICs: Interactions of People with Natural Systems and Wildlife

The effectiveness of conservation policy is dependent on its ability to influence human–environment interactions. Conservation economics in LMICs provides both empirical and theoretical models of human behavior that inform conservation policy to address anthropogenic causes of biodiversity loss, as called for by conservation scientists (Reddy et al. 2017; Dobson et al. 2019). These frameworks recognize that patterns of land use are informed by, and inform, decisions of people in LMICs that cause biodiversity loss (e.g. Pfaff 1999; Andam et al. 2008; Pfaff et al. 2009; Albers et al. 2019). Both environmental and socio-economic characteristics of LMICs inform people’s decisions and interactions with their environment and are often critical considerations in conservation policy. Governments, large scale land actors, and individual people make decisions that combine to determine the pattern of landscapes and land uses. In turn, tropical LMIC people’s land use and environmental interaction decisions consider patterns of government investments in roads and protected areas, larger scale land users, and agricultural policies (Albers et al. 2017). Here, we discuss the empirical analyses that identifies drivers of conservation outcomes, critique their ability to identify policy tools to influence individual decisions, and describe existing LMIC models of human-environmental interaction with conservation policy levers to form the basis for the next section’s discussion of incorporating disease risk in these frameworks.

Investigating deforestation, land use change, fragmentation, and park effectiveness, econometric analysis of spatial imagery and GIS datasets identifies correlations between physical and socioeconomic characteristics and conservation outcomes (e.g. Pfaff 1999; Andam et al. 2008; Pfaff et al. 2009; Busch et al. 2015; Leblois et al. 2017). Roads both create fragmentation and encourage follow-on land conversion and fragmentation (Saunders et al. 2002; Freitas et al. 2010), and protected areas and community management alter fragmentation (Sánchez-Azofeifa et al. 1999; Southworth et al. 2004; Nagendra et al. 2008; Sims 2014). Although such empirical analysis is important for identifying big-picture issues and correlations, this style of empirical analysis presents three challenges for policy to influence people’s behavior. First, such analysis obscures the incentives driving behavior and considers the actions of people only implicitly, which complicates identification of behavioral policy levers. Second, remotely sensed datasets often do not contain information about socio-economic and institutional settings that influence behavior, such as which forests are effectively managed by community organizations or which villages face costly market access (Robinson et al. 2008; Ghate et al. 2009). Third, heterogeneity of cultures, landscapes, and other characteristics of individual LMICs make extrapolating empirical results from one location to another difficult (Leblois et al. 2017), such as across settings with different acceptability of eating primate meat between Muslims and Christians (Nyanganji et al. 2011).

Conservation economics modeling work in LMICs emphasizes the role of the socio-institutional and ecological settings in which people make decisions on human–environment interactions (e.g. Gunatileke and Chakravorty 2003; Albers and Robinson 2013). In LMICs, many production and labor allocation decisions are influenced by natural environments, such as agricultural activities and non-timber forest product (NTFP) extraction, including hunting (Robinson 2016; Nielsen et al. 2017). Costly or limited market access influences decisions about how much to interact directly with natural environments for extraction and farming when facing a subsistence need or cash requirement (Robinson et al. 2002; Sills and Abt 2003; Muller and Albers 2004; Ghate et al. 2009). Decisions to consume or trade wildlife depend on market access, prices, and opportunity costs (Damania et al. 2005; Wilkie et al. 2005), in addition to other aspects of the household’s production function including protecting crops from wildlife (Johannesen and Skonhoft 2004; Bulte and Rondeau 2007). Lack of clearly defined or enforced property rights create incentives that result in de facto open access use and habitat degradation (Bulte and Engel 2006; López-Feldman and Wilen 2008). Models that incorporate spatial decisions—about extraction and land use—provide a more explicit representation of the human–environment interactions that drive conservation outcomes and reflect the impact of property rights, community management, resource density, and market access on those interactions (Robinson et al. 2002, 2008; Albers et al. 2019). These economic decision models provide a lens through which to interpret data correlations and highlight policy levers for steering human interactions with the environment. Developing general models of people’s decisions within their social and ecological context, augmented with appropriate observation and data, can help form context-specific policy that focuses on changing people’s behavior in pro-conservation ways that data analysis identifies as policy priorities.

## Expanding Models to Better Inform Joint Conservation and Zoonotic Disease Policy

Economists, health scientists, and conservation actors alike call for more research and policy that simultaneously addresses conservation and disease risk (Salkeld et al. 2013; Civitello et al. 2015; Pattanayak et al. 2017). Often, conservation policy mitigates disease risk simply because conservation policy reduces contact between people and natural environments and wildlife.^1^ Deforestation, land use change, and fragmentation all correlate with both biodiversity loss and zoonotic disease risk (Patz et al. 2004; Suzán et al. 2008; Morand et al. 2019). Ecological models predict the geographic locations where new diseases may emerge or from which wildlife species they may emerge, but, despite including demographic variables, these models do not explicitly consider the interactions of people with their environment, which is a factor in the risk of zoonotic disease transmission (Allen et al. 2017; Olival et al. 2017). That transmission occurs through a combination of environmental, epidemiological, and human behavioral factors: first, human decisions determine the characteristics of the ecological landscape; second, the resulting environmental conditions determine the distribution of, and intensity of infection in, reservoir hosts; and third, the choice of activity and amount of time allocated to specific activities (hunting, farming, harvesting NTFPs) drive the risk of exposure to pathogens (Plowright et al. 2017). As an example, the likelihood of mosquito-borne pathogen transmission depends on the abundance of mosquito vectors on a landscape, the pathogen load within the mosquito population, the chance that a pathogen-carrying mosquito bites a person, and the probability that the pathogen successfully infects the human (Baeza et al. 2017).

To depict risks on the landscape, disease ecologists create global emerging infectious disease “hotspot” maps that correlate past emerging infectious disease events, demographic data, and environmental variables to estimate the spatial relative risk of zoonotic spillovers worldwide. These models were developed at a coarse spatial resolution due to the sparsity of the data and multi-scale variables (Allen et al. 2017). Human decisions around resource use and environmental interactions occur at a finer scale, making those interactions difficult to represent in this framework despite their critical role in determining the risk of disease. To address how humans influence zoonotic disease risk borne from environmental interactions, these hotspot maps can be combined with economic decision models at fine resolution that specify markets and institutions, landscape patterns, and resource use in LMICs, and thereby illustrate the decisions behind where and how people overlap with pathogen hosts, which influences their pathogen exposure.

Using conservation economics models as a basis for developing policy that addresses both conservation and disease risk requires modeling extensions and new information. First, because both the amount of time and the locations of human–environment interactions contribute to disease risk within the ecological setting, expanding models of decisions about agricultural expansion, wildlife hunting and marketing, and resource extraction to incorporate space and time as decision variables provides a mechanism for characterizing the disease risk of such actions and for policies to internalize the disease risk into decisions. Second, because both the ecological landscape of pathogens and human activities vary seasonally, economic models at the sub-annual level could identify seasonally targeted policies to mitigate disease risk. Third, although empirical analysis signals the role of fragmentation in both biodiversity loss and zoonotic disease incidence, little ecological, economic, or interdisciplinary research depicts the process of fragmentation in a manner that helps define policy tools that influence people’s actions in ways that could limit conservation losses and disease risk from fragmentation.

In addition to such extension of modeling frameworks to address disease risk in the LMIC context, we propose an interdisciplinary and iterative process of on-the-ground observation; economic, behavioral, and epi-ecological modeling to guide further field work; and data collection in a standardized manner to inform further research and policy efforts. That iterative process might begin with observation, both finding correlations in data and through stakeholder input; followed by economic analysis of models of human decisions parameterized with empirically-relevant values to identify the critical mechanisms and drivers of the human—environment interaction and potential for policy; followed by prioritization of ecological or economic data collection to form policy in specific settings; and an iteration back to broader data analysis to discern policy impact. Such interdisciplinary analysis incorporating economic modeling will improve our ability to define factors that influence people’s decisions involving interaction with the environment and the related disease risk. In the next two sub-sections, we discuss how to extend economic models of environmental interactions in LMICs to incorporate disease risk in settings in which the risk is directly related to the actions taken by people, such as wildlife marketing, and in settings in which the disease risk derives indirectly or incidentally from the environment, such as siting livestock operations at the forest margins.

### Modeling Direct Interactions with Disease Risk

The trade in legal and illegal wildlife is worth billions of dollars, with hundreds of millions of animals and plants traded globally (Fukushima et al. 2020). Wildlife trade creates a direct human-wildlife interaction through intentional contact with the live or dead wildlife species at any point along the supply chain—from hunting to trading and selling to consumption (Daszak et al. 2000; Kruse et al. 2004; Breed et al. 2006). For example, SARS-COV and SARS-2-COV are linked to wildlife trade in China, HIV to primate bushmeat hunting, monkeypox virus to the exotic pet trade, and H5N1 and H7N9 to domestic poultry (Karesh et al. 2005; Gilbert et al. 2014; Andersen et al. 2020). Capturing, raising, and transporting live animals brings many individuals into close proximity, creating opportunities for inter- and intra-species pathogen transmission and potentially spillover to humans. For example, SARS originated in wet markets where the pathogen spilled over from bats to civets (Li et al. 2005), and SARS-2-COV may have originated with pangolins as an intermediary host (Andersen et al. 2020). In these cases, the lack of regulations and poor biosafety at nodes on the supply chain increased the risk for vendors and purchasers further downstream.

As the starting point of the wildlife supply chain, hunting activity that supplies markets poses a risk of disease spread as hunters interact with live animals (Wolfe et al. 2005). Given that interaction, the drivers of hunting decisions can be targeted by policy to reduce disease risk and promote conservation. Households face tradeoffs in their decisions to allocate time or land between agriculture and wildlife harvesting (Bulte and Horan 2003). When these households also consume harvested wildlife, the income elasticity of demand for bushmeat drives the response to an alternative livelihood policy, identifying this elasticity as priority data (Damania et al. 2005). Response to policy may also differ when the hunted species are considered a nuisance to agriculture and are taken to defend crops as well as for consumption or sale. This dual purpose of hunting affects the tradeoffs hunters face when reacting to policy (Johannesen and Skonhoft 2004; Bulte and Rondeau 2007).

Empirical analyses find variation in the response of hunters to the relative costs of hunting in an environment, such as park proximity; labor tradeoffs with other activities, particularly livestock; and household characteristics (e.g. Brashares et al. 2011; Foerster et al. 2012; van Velden et al. 2018). Hunters may respond to the increased opportunity cost of hunting by switching to more efficient hunting techniques and more valuable species, which does not address disease risk and presents a conservation risk when those valuable species are protected species (Damania et al. 2005). When hunters cannot discriminate in the species they hunt, enforcement policies may have unintended consequences around consumption of protected species (Robinson 2008). Hunters’ decisions rarely include disease risk mitigating activities, perhaps due to a lack of knowledge of the risk the animal presents (Harrison et al. 2011).

Policies to alter incentives for bushmeat hunting and consumption include enforcement of illegal hunting, community-based conservation, and alternative or additional livelihoods and protein sources (Foerster et al. 2012; van Velden et al. 2018). Models predict the outcomes of these policies, showing in what circumstances alternative livelihoods, enforcement, and expanding protected areas lead to negative conservation outcomes (Damania et al. 2005; Johannesen 2007; Robinson 2008). Policies that reduce hunting itself create conservation and disease risk mitigation but can impose economic burdens on hunters and demand for wildlife/bushmeat continues to create incentives for hunting. Policies that promote safer hunting practices, information about disease risk, and incentives to hunt lower risk species directly alter the disease risk within the context of ongoing hunting.

Between the hunters and the final markets, traders, middlemen, and sellers in the wildlife/bushmeat supply chain serve a crucial role in LMICs by providing access to markets, and also face disease risk. Yet, few economic models and empirical analyses include the role of these other actors, which misses additional points of human-disease interaction that could respond to policy (Bowen-Jones et al. 2003; Cowlishaw et al. 2005; Kamins et al. 2011; Nielsen et al. 2014; Bachmann et al. 2019; Van Vliet et al. 2019; Latinne et al. 2020). Middlemen with sufficient market power can pay lower prices to hunters than middlemen operating competitively and prevent the system from reaching the open access outcome (Tháy et al. 2019). This role of middlemen is relevant in modeling wildlife supply chains and the resulting disease externality because they drive two elements of the disease risk: the amount of wildlife being harvested and the number of people directly contacting the wildlife. All points of human-wildlife contact along the supply chain represent areas of zoonoses spillover risk and possible opportunities for changing human behavior with policy levers.

Both local and global consumers contribute to the demand for wildlife and bushmeat (McNamara et al. 2019; Fukushima et al. 2020). Local demand in LMICs is largely driven by wealth, nutritional needs, and prices (Wilkie et al. 2005; Fa et al. 2009; Godoy et al. 2010). Analysis of demand across countries finds a range of income elasticities and price elasticities for bushmeat (Wilkie and Godoy 2001; East et al. 2005; Wilkie et al. 2005; McNamara et al. 2019). Policy can be aimed at manipulating the prices of bushmeat to reduce demand, and thereby reduce disease risk to consumers and the supply chain by reducing supply, but variation across settings in demand response imply a need for context-specific information.

### Example: Sulawesi Wildlife Markets Supply Chain

Here we demonstrate the role of balancing observation and data collection with modeling of people’s decisions around their interaction with wildlife to inform conservation and disease policy. The wildlife markets in North Sulawesi, Indonesia sell a variety of wildlife and domestic animals, including bats like large flying foxes, which could potentially lead to disease spillover events. Bat sales in these markets have been increasing for decades (Clayton and Milner-Gulland 2000; Lee et al. 2005; Latinne et al. 2020) and concerns about declining bat populations and potential pathogen spillovers led to calls for policy to reduce bat harvesting (Sheherazade and Tsang 2015; Latinne et al. 2020). Latinne et al. (2020) conducted stakeholder interviews with hunters, local collectors, middlemen, and vendors, and collected market data during 2016–2019 to describe the supply chain and estimate quantities of marketed bats. An important feature of these markets is that regional resource use and cultural differences between geographic regions create the current structure of the supply chain. Demand for bat protein stems from Christians in northern Sulawesi, where hunting has historically occurred and reduced bat populations. Current supply is transported from provinces in southern Sulawesi, which are predominantly Muslim and do not consume bats, and therefore have larger populations to hunt (Clayton and Milner-Gulland 2000; Lee et al. 2005; Sheherazade and Tsang 2015). Hunters sell the bats to either to local collectors or directly to middlemen, who transport the bats to the markets in the north (Latinne et al. 2020).

By identifying the actors and collecting data on revenues and costs, Latinne et al. (2020) identifies two salient characteristics of this market for policy development—hunters operate under open access conditions and middlemen transport animals to markets, factoring transportation costs into prices. Combining observations, stakeholder interviews, and data collection, followed by modeling the entire supply chain, highlights specific incentives to target in order to conserve bats and mitigate pathogen spillovers from wildlife. The construction of the Trans Sulawesi Highway in 1980 allowed vendors to travel further to collect wildlife and expanded the region over which bat harvest creates conservation losses and disease risk (Clayton and Milner-Gulland 2000). Policies such as highway tolls that increase transportation costs to middlemen could reduce the volume of bats traded because hunters cannot readily access markets. In this case, data collection on the elasticities of demand for bat meat would be a critical piece of information to collect to determine whether the middlemen may pass on the price increase to consumers. Second, policies aimed at providing alternative income sources to hunters could increase middlemen costs and reduce both harvest and disease risk. The policy could also increase the practice of middlemen transporting hired hunters to harvest the area, and disease risk and conservation concerns would continue (Latinne et al. 2020). To counter coordination between middlemen and hunters, policies that provide incentives for people in southern Sulawesi to both forego hunting and protect bats from hunting could prevent these migrant hunters from accessing bat habitat. Despite the valuable ecosystem services provided by bats, including fruit pollination, bats are generally considered a nuisance species by fruit farmers on Sulawesi, which limits the farmers’ interest in providing such incentives to bat hunters (Banack 1998; Latinne et al. 2020). Closer collaboration between conservation and health organizations could support payments for ecosystem services to potential bat hunters to discourage bat harvest and disease risk while promoting bat conservation for bat pollination and seed dispersal services.

The iterative process of field work and modeling the supply chain, informed by stakeholder interviews, establishes a fuller picture of the disease externality risk at each contact point and the decisions of all actors and forms the basis for policy analysis aimed at reducing human-wildlife contact’s disease risk and promotes conservation.

### Modeling Indirect Interactions with Disease Risk

Some risk of disease arises through frequent human interactions with the environment in which contact with wildlife hosting a pathogen is incidental or an unintended consequence. These types of environmental interactions tend to drive persistence of endemic infectious diseases. For example, while harvesting fuelwood or other NTFPs, individuals increase their exposure to arthropods or non-target wildlife species that are carriers of infectious pathogens like malaria and yellow fever (Barros and Honório 2015). In southeast Asia, pathogen transmitting mosquito species are found in greater abundance near deforested land and oil palm plantations (Young et al. 2020). The expansion of agricultural land, in particular oil palm and rubber in Liberia and sugar-cane in Bolivia, has increased the frequency of contact between plantation workers and rodents that are attracted to these environments, increasing thus the risk of hemorrhagic fevers (e.g. Lassa fever in Africa or Ordog Fever in South America) (Olugasa et al. 2014; Patterson et al. 2014; Gibb et al. 2017). The decision to site livestock operations near forest habitat increases the likelihood of interactions and conflicts between livestock and potential wildlife reservoirs for pathogens. For example, the emergence of Nipah virus in Malaysia was directly linked to agricultural intensification. Pig farms were established in close proximity to forest; farmers planted fruit trees for additional income with the unintended result of attracting bats, which are natural reservoirs of Nipah virus (Epstein et al. 2006). In each of these examples, the risk of disease is endogenous in the decision to interact with the environment, but the risk is incidentally related to that decision. In these settings, the context of when, where, and how individuals interact with the environment plays an important role in the risk of disease.

Indirect interactions with disease risk are driven by choices individuals make about how and where they spend their time with respect to activities such as subsistence farming, collecting NTFPs, mining, or working in the agricultural sector. These choices affect the land covers, habitat degradation, and wildlife with which individuals interact. As an example of a modeling opportunity, the ecological literature indicates that disease risk is greatest in altered or degraded natural habitats (Murray and Daszak 2013), which could link to people’s decisions as a function of resource quality. Modeling the choice of labor allocation within a particular socio-institutional setting explicitly allows for representing trade-offs of allocating time to specific activities across land covers. Labor allocation results can be combined with ecological models or data on vector or pathogen abundance across landscapes to determine individual pathogen exposure risk. The setting’s institutions can also influence disease risk. For example, introducing markets for fuelwood substitutes alters the labor allocation to that environmental interaction and exposure to pathogens. Similarly, establishing community property rights in forests that have been degraded by over-extraction in de facto open access mitigates disease risk by reducing pathogen prevalence and reducing extraction within the forest. With more contextual information about activity choices from socio-economic field work, the daily and seasonal timing of activities can further inform infection risk. For example, if a wildlife species has crepuscular activity and agricultural work is conducted in the cool early morning, high vector activity and human presence on the same landscape may create a high-risk setting for pathogen transmission (Vittor et al. 2006).

Policy to reduce the risk of disease transmission through indirect interactions can take one of two approaches, either limiting resource extraction or limiting exposure to pathogens in risky environments. Limiting the extent of extractive industries can simultaneously address conservation and health policy goals, but limiting opportunities for economic activities can result in illegal resource extraction, and disease incidence is often greater around illegal resource extraction operations (Castellanos et al. 2016). To limit extractive industries would therefore require creating alternative (legal) economic opportunities or payment for ecosystem service programs (Cárdenas 2017) and monitoring and enforcing regulations. Alternatively, policies can be developed using information about which specific actions increase human contact with the pathogen, and therefore create the greatest risk of pathogen transmission. For example, if oil palm plantation workers live near fields, increasing their risk of exposure to rats and Lassa fever, housing can be sited in locations farther away from the source of risk. Finally, disseminating information about how specific activities increase disease risks may be a viable policy to mitigate pathogen transmission.

### Example: Malaria Risk and Labor Allocation Decisions Across Time and Space

Here, we explore an example of how leveraging empirical findings and expanding existing models, in combination with socio-economic and environmental observations, can inform policy to reduce malaria risk. A majority of the empirical disease research examines disease incidence from incidental interaction between humans and disease vectors stemming from economic activities such as land use change. Ecological research suggests that deforestation opens the forest canopy, which modifies moisture and sunlight that reaches the ground and creates ideal breeding conditions for mosquitoes, which can transmit malaria (De Castro et al. 2006; Vittor et al. 2006, 2009). Simultaneously, deforestation reduces the abundance of species that feed on mosquitoes, amplifying both the density of the vector and pathogen (Baeza et al. 2017). In the Amazon region of South America, the malaria vector species *Anopheles sp.* occurs in greater abundance at forest edges and agricultural landscapes and in lower densities in forests and protected areas; *Anopheles* abundance and diversity varies across land cover types (space); and *Anopheles* feeding activity varies by time of day and season (Stefani et al. 2013; Bauch et al. 2015). These findings imply that where and when people choose to perform activities is important in exposure to pathogens and disease risk. Combining evidence from the natural sciences with economic models that explicitly represent when and where individuals spend time between, for example, collecting NTFPs in a protected area and working in agricultural fields can provide insight into how incentives shape risk of infectious disease.

Existing econometric studies relating deforestation to malaria incidence have been conducted for South America, Africa, and Asia, and rely on geospatial datasets in combination with either regional epidemiological data (Valle and Clark 2013; Hahn et al. 2014; Terrazas et al. 2015; Garg 2019) or individual health survey data (Berazneva and Byker 2017; Bauhoff and Busch 2020). While the majority of studies find either positive correlation or causation between deforestation and disease incidence, these results are not consistent across all published works (e.g. Valle and Clark 2013; Bauhoff and Busch 2020). Although the increasing availability of geospatial and large datasets provides insight into global trends and hotspots for diseases, how people’s decisions contribute to the risk of disease spread cannot be discerned from data at the parcel or pixel unit of analysis. As a result, the policy implications and ability to generalize results from these empirical studies are limited to identifying specific demographics that can be targeted for malaria prevention and control programs and broad statements about curbing rates of deforestation.

As one possible starting point, Albers et al. (2019)’s model of labor time allocation between wage labor and NTFP extraction incorporates both an individual spatial extraction path decision and an aggregate spatial equilibrium of NTFP extraction in a forest. That framework could be expanded to replace wage labor with agriculture, to further refine the timing and seasonality of decisions, and to reflect a range of socio-institutional and ecological settings. This modeling framework can be validated and parameterized using stakeholder interviews and observational data. Then, overlaying this economic spatial land use model with ecological models that determine mosquito bite rate probabilities in agricultural land, fringe forest, deeper forests, and protected areas allows the model’s solution to define the malaria transmission risk facing each individual based on their where and when they decide to perform specific activities. Analyzing the spatial pattern of resource extraction decisions as a function of ecological, socio-institutional, and market settings can reveal the characteristics of settings and individuals that drive malaria risk and provide insight for policy development. Simultaneously considering the daily and seasonal timing of human and vector activity (mosquitoes are most active at dawn and early evening, and most abundant around rainy seasons (De Castro et al. 2006)) can avoid creating policies that unintentionally increase pathogen transmission risk. For example, creating alternative fuel sources may decrease the total time spent in forests extracting fuelwood but focus that reduced time in NTFP extraction in fringe forests that have higher mosquito abundance, thereby increasing disease risk. Joint consideration of the impact of policies on conservation and health outcomes can avoid perverse incentives and unintended outcomes. Supplementing sensitivity analysis with stakeholder observations and data to describe particular settings produces stronger understanding of the drivers of decisions that create the disease risk and identifies the information and data necessary to provide the right policies for the ecological and institutional setting, across seasons.

## Call for Next Steps

Environment, resource, and development economists are particularly well-suited to developing frameworks of people’s decisions to interact with their environments; and such models can identify policy levers that alter people’s actions in ways that promote both conservation and disease risk mitigation. We suggest three guides for policy-relevant research:

*Data analysis to identify correlations between land/resource characteristics and disease risk is necessary and important but not sufficient to guide joint conservation and disease-risk mitigating policy.* Empirical analysis that defines correlations but is not specific to people’s decisions does not provide information about how human–environment interactions affect conservation or disease spread, which implies that policy levers are difficult to identify below generalities, such as “slow deforestation,” “limit fragmentation,” and “close wildlife markets.”

*Modeling people’s decisions to interact with natural resources in a LMIC setting, while using an iterative process that incorporates context and data, enables policy design based on people’s decisions and disease spread mechanisms that can be modified for specific settings.* Because empirically-informed models characterize decisions about interactions with the environment, they can be used to identify how people react to policies that drive both conservation and disease risk outcomes. In the absence of detailed data at every site of conservation and disease policy interest, general models can prioritize particularly important data collection to enable the model’s application in other eco-institutional settings.

*Although economic models of people’s decisions to interact with the environment are important for defining conservation-disease policy, interdisciplinary research with ecologists and epidemiologists is necessary to accurately reflect the mechanisms of disease spread that matter for policy.* The risk of disease spread is a function of people’s choices and of how those choices lead to pathogen exposure, which implies that economic models must be paired with data and models of those disease risks. Economic models of human-resource interaction that differentiate across choices of locations and time spent, and across seasons, may be necessary to characterize human infection risk. In turn, economic policy analysis can prioritize further research into ecological mechanisms that drive risk in specific environments.

## Abbreviations

LMIC: Low and middle income country
EID: Emerging infectious disease
NTFP: Non-timber forest products

## Footnotes

1 For example, regulating bushmeat consumption would reduce human contact with wildlife through hunting, thereby conserving the species and mitigating pathogen spillover risk. Policies that directly target disease risk, however, do not necessarily create a conservation benefit. For example, a policy to enhance biosecurity in handling wildlife does not mitigate wildlife hunting beyond increasing costs. Similarly, a policy to provide information to people about disease risk in the landscape may induce people to alter their patterns of environmental interaction in ways that negatively impact conservation outcomes.
